# How autoimmune antibodies kindle a firestorm in the brain

**DOI:** 10.1038/s44319-024-00094-w

**Published:** 2024-02-28

**Authors:** Johannes W Hell

**Affiliations:** Department of Pharmacology, University of California, Davis, CA 95616-8636 USA

**Keywords:** Immunology, Molecular Biology of Disease, Neuroscience

## Abstract

Patient-derived autoantibodies against NMDARs and GABAaRs show a crossover effect on the opposite receptor’s localization and function dependent on neuronal activity.

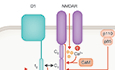

## Teaser

Autoimmune responses against NMDA receptors (NMDAR) and GABA_A_ receptors (GABA_A_R) can cause encephalitis, which has been described as feeling like one’s brain is on fire (Dalmau et al, 2019). The molecular mechanisms of how the autoantibodies affect neuronal functions are incompletely defined. Hunter et al (2024) now describe effects of autoantibodies from patients against NMDARs and GABA_A_Rs on the lateral mobility and thereby localization and function of NMDARs and GABA_A_Rs as well as AMPA receptors (AMPARs), the main glutamatergic receptors that mediate basal synaptic transmission. The study provides evidence for crossover effects of autoantibodies on the opposite receptor that depend on neuronal activity and the interaction of NMDAR with the dopaminergic receptor D_1_.

AMPAR mediate basal synaptic transmission at excitatory synapses and NMDAR the Ca^2+^ influx that is triggered by heightened synaptic activity and in turn induces plasticity changes at individual synapses such as long-term potentiation (LTP) and long-term depression. GABA_A_R mediate synaptic transmission at inhibitory synapses that suppress neuronal activity. In this way, AMPAR and NMDAR activity on one hand and GABA_A_R activity on the other hand determine the balance between excitation and inhibition in the brain (Ghatak et al, 2021). Using cultured hippocampal neurons, Hunter et al (2024) found that autoimmune antibodies against either NMDARs or GABA_A_Rs have similar effects on the mobility of both receptors in the plasma membrane, decreasing the diffusion of NMDARs and increasing it for GABA_A_Rs. Despite the opposite effects on diffusion, the final outcome was the same for both kinds of autoantibodies: a decrease in the number of clusters of NMDARs and GABA_A_Rs. In addition, cluster size was reduced for NMDARs and AMPARs but not for GABA_A_Rs. Inhibition of neuronal activity with tetrodotoxin inhibited the effect each autoantibody had on the other receptor but not on its target receptor, i.e., it mitigated the effect of NMDAR antibodies on GABA_A_R but not NMDAR cluster density. Likewise, the effect of GABA_A_R antibodies on NMDAR but not GABA_A_R cluster density was prevented.

Electrophysiological analysis showed a reduction in the amplitudes of AMPAR-mediated spontaneous excitatory postsynaptic currents (sEPSCs), which is consistent with the reduction in AMPAR cluster size. This effect was again observed for autoantibodies against NMDARs as well as GABA_A_Rs. A similar albeit more modest reduction in spontaneous inhibitory postsynaptic currents (sIPSCs) was also observed. The overall effect on neuronal activity was a pronounced shift in the excitation/inhibition balance towards excitation, a common denominator for a number of brain disorders such as schizophrenia, autism-spectrum disorders, Rett syndrome, and Alzheimer’s disease (Ghatak et al, 2021).

Using a phosphoproteomic approach, Hunter et al (2024) identify several kinases whose activities are affected by the autoantibodies. The activity of glycogen synthase kinase-3 (GSK3) was increased by both types of autoantibodies. It is notable in this context that GSK3 is required for NMDAR-dependent long-term depression of synaptic transmission in the hippocampal CA1 region, which is mediated by a reduction of postsynaptic AMPAR content (Peineau et al, 2007). Activation of GSK3 could, thus, be involved in the decrease in postsynaptic AMPAR activity induced by both types of antibodies. Because GSK3 binds to casein kinase 1 (CK1), which can serve as a priming kinase of several GSK3 substrates, Hunter et al (2024) tested whether inhibition of CK1 (with the organic compound CK17) would affect the autoantibody effects. CK17 prevented the effect of the GABA_A_R antibody on NMDARs.

**Figure 1:**
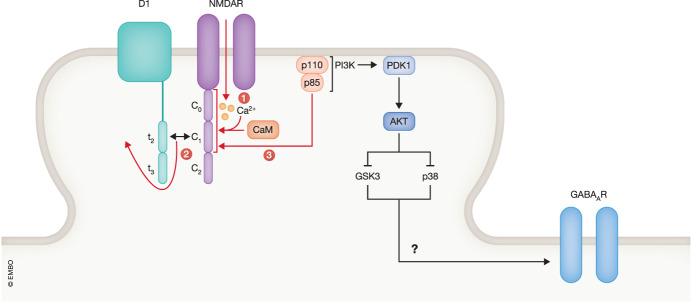
Hypothetical interplay between neuronal activity and autoantibodies against NMDARs. Neuronal activity induces Ca^2+^ influx via NMDARs. Red arrows depict binding of Ca^2+^ to calmodulin (CaM) and of Ca/CaM to the GluN1 C0 and C1 regions, and the resulting displacement of the D1 t2 region from C0, which allows binding of PI3K consistent of the P110 and p85 subunits to bind to GluN1. This interaction might recruit PDK1 and ACT (aka PKB) to NMDAR - rich subdomains, thereby sequestering those kinases away from p38 and GSK3. This sequestration could disinhibit GSK3 and p38, which in turn could signal to GABA_A_R, although none of these latter effects downstream of Akt have been identified.

Autoantibodies might affect interactions of NMDARs and GABA_A_R with other proteins. NMDAR localization is regulated by its association with the dopaminergic receptor D_1_ (Petit-Pedrol and Groc, 2021). The C1 segment in the cytosolic C-terminus of the NMDAR GluN1 subunit binds to the t2 segment in the cytosolic C-terminus of D_1_ (Lee et al, 2002; Pei et al, 2004) (Fig. 1), Hunter et al (2024) evaluated whether manipulating this interaction would influence the autoantibody effects. Ectopic expression of D_1_, in which t2 was deleted, abolished the effect of the NMDAR antibody on GABA_A_Rs but not NMDARs and of the GABA_A_R antibody on NMDARs but not GABA_A_Rs. Thus, it appears that the crossover of effects between binding of autoantibodies to NMDARs or GABA_A_Rs to the opposite receptor depends on the association of D_1_ with GluN1 while this association was not necessary for the effects of the autoantibodies onto their respective target receptor. This dependency of the crossover effects on D_1_ binding to GluN1 is paralleled by its dependency on neuronal activity (see above) but whether and how D_1_ is affected by the autoantibodies and how this activation and neuronal activity mediate the crossover effects is unclear.

Activity-driven Ca^2+^ influx through the NMDAR might be one mechanism that could link neuronal activity to the D_1_—GluN1 interaction and their role in the crossover effect. Ca^2+^ influx governs the interaction of the universal Ca^2+^ sensor calmodulin (CaM) with the GluN1 C-terminus (Figure) (Fig. 1, step 1) (Merrill, 2007). Ca^2+^/CaM displaces D_1_ from the GluN1 C-terminus (Fig. 1, step 2), which leads to recruitment of the PI3 kinase (PI3K) p85/p110 to GluN1 and PI3K activation (Fig. 1, step 3) (Lee et al, 2002). PI3K can activate a myriad of signaling cascades, many via PDK1 - AKT/PKB signaling but how any of these cascades relate to the autoantibody effects is a wide open question, especially as AKT inhibits GSK3 (Hemmings and Restuccia, 2012) rather than activates it as would tie in with the increase in GSK3 activity that is observed with the autoantibodies Hunter et al (2024). Like GSK3, the mitogen-activated protein kinase (MAPK) p38 has been implicated in LTD (Zhang et al, 2018; Zhu et al, 2002) and could also be involved in mediating the reduction in AMPAR by the anti-NMDAR autoantibodies. However, like GSK3, p38 seems in general downregulated by AKT activity (Rane et al, 2010). Perhaps, the exact up or downregulation of GSK3 or p38 could depend on subcellular domains that exist even within the limited space of dendritic spines, the postsynaptic sites of glutamatergic synapses, as is the case for p38 functioning within the lysosomal compartment (Zhang et al, 2018). Changes in this compartment have recently been linked to loss of long term potentiation (LTP) (Chen et al, 2023). It is possible that binding of PI3K to GluN1 sequesters it and perhaps AKT away from p38, thereby reducing an inhibitory effect on p38.

The receptor tyrosine kinase Ephrin B receptor EphB2 stimulates postsynaptic accumulation of NMDARs by binding via its extracellular domain to the extracellular domain of GluN1 (Dalva et al, 2000; Hanamura et al, 2017). This interaction, thus, constitutes another potential target site for autoantibody interference that might be worth testing now. In addition, autoantibodies could exert so-called non-ionotropic effects as have been emerging over the past two decades for some actions of NMDAR agonists (Park et al, 2022).

Another open question is whether the majority or only a relatively small subgroup of autoantibodies against NMDARs and GABA_A_R affect receptor mobility. The authors only tested two different monoclonal autoantibodies against the NMDAR and two different ones against the GABA_A_R, with similar results on density and area of NMDAR and GABA_A_R clusters. More autoantibodies need to be tested to see whether these effects are seen with most autoantibodies and perhaps even antibodies raised by investigators that might or might not have the neurological effects seen with the autoantibodies.

In conclusion, Hunter et al (2024) advance our understanding of autoantibody effects in autoimmune encephalitis and open new perspectives for future experiments.
